# Construction of a single nucleotide variant score-related gene-based prognostic model in hepatocellular carcinoma: analysis of multi-independent databases and validation in vitro

**DOI:** 10.1186/s12935-021-02321-z

**Published:** 2021-11-18

**Authors:** Yu-Jie Xu, Min-Ke He, Shuang Liu, Li-Chang Huang, Xiao-Yun Bu, Anna Kan, Ming Shi

**Affiliations:** 1 State Key Laboratory of Oncology in South China, Collaborative Innovation Center for Cancer Medicine, Sun Yat-Sen University Cancer Center, Guangzhou, China; 2 Department of Hepatic Surgery, Sun Yat-Sen University Cancer Center, Guangzhou, 510060 China

**Keywords:** Hepatocellular carcinoma, Single nucleotide variant, Prognostic model, Immune microenvironment

## Abstract

**Background:**

The accumulation of single nucleotide variants (SNVs) and the emergence of neoantigens can affect tumour proliferation and the immune microenvironment. However, the SNV-related immune microenvironment characteristics and key genes involved in hepatocellular carcinoma (HCC) are still unclear. We aimed to evaluate differences in the SNV-related immune microenvironment, construct a prognostic model and validate the key genes in vitro.

**Methods:**

The categories of samples were defined by the expression of SNV score-related genes to evaluate the differences in mutational features, immune environment and prognosis. The survival model was constructed with survival-associated genes and verified in two independent test datasets. RCAN2, the key gene screened out for biofunction, was validated in vitro.

**Results:**

IC2, among the three integrated clusters (IC1, IC2, IC3) classified by the 82 SNV score-related genes, was distinct from the rest in SNV score and immune cell infiltration, showing a better prognosis. Seven prognostic markers, HTRA3, GGT5, RCAN2, LGALS3, CXCL1, CLEC3B, and CTHRC1, were screened to construct a prognostic model. The survival model distinguished high-risk patients with poor prognoses in three independent datasets (log-rank P < 0.0001, 0.011, and 0.0068, respectively) with acceptable sensitivity and specificity. RCAN2 was inversely correlated with NK cell infiltration, and knockdown of RCAN2 promoted proliferation in HCC.

**Conclusions:**

This study revealed the characteristics of the HCC SNV-associated subgroup and screened seven latent markers for their accuracy of prognosis. Additionally, RCAN2 was preliminarily proven to influence proliferation in HCC and it had a close relationship with NK cell infiltration in vitro. With the capability to predict HCC outcomes, the model constructed with seven key differentially expressed genes offers new insights into individual therapy.

**Supplementary Information:**

The online version contains supplementary material available at 10.1186/s12935-021-02321-z.

## Background

Hepatocellular carcinoma (HCC) is the sixth most common cancer, with mortality ranking third for malignancies in 2020 [1, 2]. A large number of hepatocellular carcinomas are diagnosed in advanced stages and are not suitable for surgical resection. However, insensitivity to systemic chemotherapy is a troubling problem in the treatment of advanced HCC [3–5]. With the development of immunotherapy, the overall survival of patients with HCC has been prolonged, and tumour-specific neoantigens are gradually being recognized [6].

Rapid replication of DNA accompanied by mutations is considered one of the key features of the development of HCC. The generation and accumulation of somatic mutations is regarded as a driving factor for HCC and is related to tumour-specific neoantigens [7]. Interestingly, the accumulation of mutations is usually correlated with a poor survival in multiple cancers [8]. However, patients with high levels of mutations can achieve better survival in immunotherapy because of the better recognition by their immune system of tumour-specific neoantigens [9, 10].

Single nucleotide variants (SNVs) is an important component of somatic mutations and some genes with SNVs can alter the HCC cells behaviours or clinical characteristics [11, 12]. In addition, the SNVs level of circulating tumour DNA (ctDNA) provides a better evaluation of HCC patients’ prognostic and tumour occurrence detection in advance than traditional strategies [13]. Therefore, focusing on SNVs could provide a new perspective for the diagnosis and treatment of HCC.

To date, there are few reports on the SNV-associated microenvironment and key genes. In this study, we screened SNV score-related genes whose expression was correlated with SNVs and classified the samples by their SNV score-related gene expression pattern. The mutation characteristics, immune microenvironment differences and overall survival among the subgroups were assessed. In addition, the biological functions of differentially expressed genes among the subgroups were further studied to explore the possible pathways and mechanisms by which SNV contributes to the prognosis of HCC. Moreover, a survival prediction model was constructed from the key differentially expressed genes and then verified by two independent test datasets. Finally, we validated the biological function of RCAN2 in vitro since it is considered one of the key genes.

## Methods

### Data collection and pre-processing

We downloaded the latest clinical data, SNV (MuSE Variant Aggregation and Masking), and RNA-seq (HTSeq-FPKM) data from the Cancer Genome Atlas–Liver hepatocellular carcinoma (https://portal.gdc.cancer.gov, TCGA-LIHC) on March 10, 2021 [14]. Patients without complete clinical data were filtered out. In addition, only genes with nonzero expression in more than 80% of HCC patients were included for analysis. Before the analysis, RNA-seq (HTSeq-FPKM) data was pre-processed by the formula as log2(fpkm + 1).

### Identification of SNV score-related genes and integrated clusters

Through the downloaded SNV data, the ‘SNVs-total’ was used as the SNV score of each patient for subsequent analysis. The Spearman correlation coefficients were calculated between the genes expressions and SNV scores of HCC patients. On this basis, related genes were screened out with a threshold of P < 0.05 and R > 0.2 and called SNV score-related genes. Based on the expression profiles of the SNV score-related genes, the samples were classified into different integrated clusters (ICs) by a clustering algorithm (R package: ConsensusClusterPlus, version: 1.52.0), with an appropriate K-means for the initial cluster [15].

### Differences in integrated clusters were assessed in different dimensions, including SNVs and clinical characteristics

At the same time, clinical data and SNVs of the samples were included to evaluate the differences in clinical characteristics, prognosis and SNV among the integrated clusters. On the basis of the above, we combined the original integrated clusters with no difference in SNV score and clinical characteristics into a new integrated cluster.

### Difference analysis of the immune microenvironment, transcriptome and biological function among new integrated clusters after incorporation

The abundances of twenty-two kinds of immune cells, naive B lymphocytes, CD4 T cells and natural killer cells (NK cells), were calculated for the integrated clusters by an approach called CIBERSORT [16]. To evaluate the infiltration of immune cells accurately, P < 0.05 was used as a threshold to judge the result of deconvolution.

In the dimensions of transcriptome analysis among the new integrated clusters, we used the limma package (version: 3.44.3) to identify differentially expressed genes that met two criteria simultaneously (1. adjusted P value < 0.05; 2. Log foldchange > 1 or < − 1) [17]. The differentially expressed genes selected above were used for the analysis of biological function in the next step. Gene Ontology (GO) enrichment analysis and Kyoto Encyclopedia of Genes and Genomes (KEGG) pathway analysis were performed for the differentially expressed genes by the clusterProfiler package (version: 3.160) [18–20].

### Identification of the key differentially expressed genes related to the prognosis and construction of the survival prediction model

To identify the key differentially expressed genes related to prognosis, Cox hazard analysis with P < 0.05 as a significant standard was performed for the differentially expressed genes screened out above, and the TCGA-LIHC dataset was used as the training dataset. We did not add other covariates into the workflow because of the lack of clinical characteristics in TCGA-LIHC database that were recognized as the influence factors of the overall survival such as BCLC stage, microvascular invasion, AFP and so on. Then, we established a survival prediction model from the key differentially expressed genes and calculated each HCC patient’s risk score with the model. All patients were divided into high-risk and low-risk groups with a risk score cut-off of 1.0. Kaplan–Meier (KM) survival curves and receiver operating characteristic (ROC) curves of the different risk groups were generated to evaluate the new survival model. The correlation between the model and SNV score was calculated as well.

### Verification of the survival model in two independent external test datasets

Two independent external test datasets were analysed to verify the rigor and accuracy of the new survival model as described above. The first external test dataset, mainly including HCC patients with surgical treatment, is available in NODE (https://www.biosino.org/node, ID: OEP000321) [21]. The second external test dataset with advanced HCC patients undergoing transcatheter arterial chemoembolization (TACE) treatment can be accessed in the Gene Expression Omnibus (https://www.ncbi.nlm.nih.gov/geo, ID: GSE104580).

### Screening out the most biofunction-correlated gene and validating its biological function in vitro

The gene most closely associated with biological function was screened out for validation in vitro. The protein expression level and the location of the protein in liver tissue were investigated by immunohistochemistry (IHC) and multicolour immunofluorescence (IF). All antibodies were obtained from the Proteintech Company, and every clinical tissue specimen was accessed after obtaining institutional review board approval.

In addition, the gene was overexpressed and knocked down in two human hepatocellular carcinoma cell lines (MHCC97H and Hep3B). MHCC97H cell was obtained at Liver Cancer Institute, Fudan University and Hep3B cell was purchased from the National Collection of Authenticated Cell Cultures (https://www.cellbank.org.cn/). All cells were maintained in high glucose Dulbecco’s modified Eagle’s medium (DMEM, Gibco) supplemented with 10% foetal bovine serum (FBS, Gibco), and their growing environments were 37 °C with 5% CO_2_. Cell proliferation was detected at days 0, 1, 2, 3 and 4 with a cell counting kit-8 (CK04, DOJINDO).

## Results

### Three original integrated clusters were identified by eighty-two SNV-related genes, and IC2 was significantly different from IC1 and IC3 in the dimensions of the SNV score and clinical characteristics

**Fig. 1:**
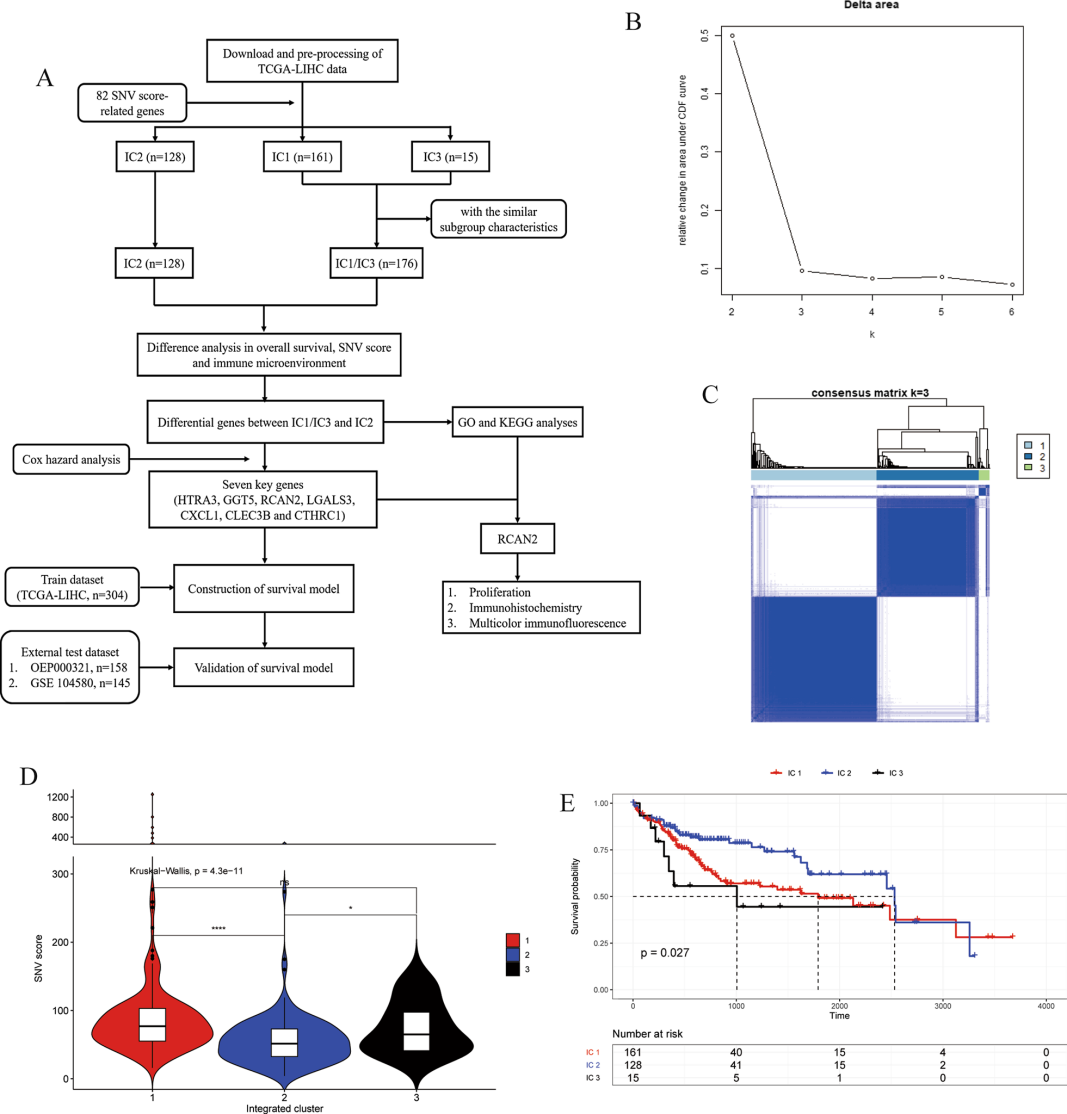
**A** The workflow of the study. **B** and **C** Results of the clustering algorithm and the integrated clusters with K = 3. **D** Differences in SNV scores among three original integrated clusters (*P < 0.05, **P < 0.01, ***P < 0.001, ****P < 0.0001, ns means no statistical significance, according to two independent samples with a Wilcox test). E, KM curve analysis of the three original integrated clusters

The analysis workflow is shown in Fig. 1A. Eighty-two SNV score-related genes were identified with two simultaneously met thresholds, including P < 0.05 and R > 0.2. According to the expression of the SNV score-related genes, 304 patients with complete clinical data were divided into three integrated clusters by a clustering algorithm. To optimize the results of the cluster analysis, we set the max groups as six and repeated them 1000 times. The results showed that the optimal cluster was three, including IC1 (n = 161), IC2 (n = 128) and IC3 (n = 15) (Fig. 1B, C).

There were significant differences in SNV scores among IC1, IC2 and IC3 (Kruskal–Wallis P < 0.05). The SNV scores of IC1 and IC3 were higher than that of IC2 (P < 0.0001 and P < 0.05, respectively), while there was no significant difference between IC1 and IC3 (Fig. 1D). The overall survival evaluated by KM survival curves among the three subgroups was significantly different (P = 0.027, Fig. 1E). Moreover, there were no significant differences among the three subgroups for clinical characteristics, including pathologic stage, pathological T stage, sex and body mass index (BMI) (Additional file 1: Table S1).

### Differences between IC2 and IC1/IC3 in overall survival, SNV score and immune microenvironment

**Fig. 2:**
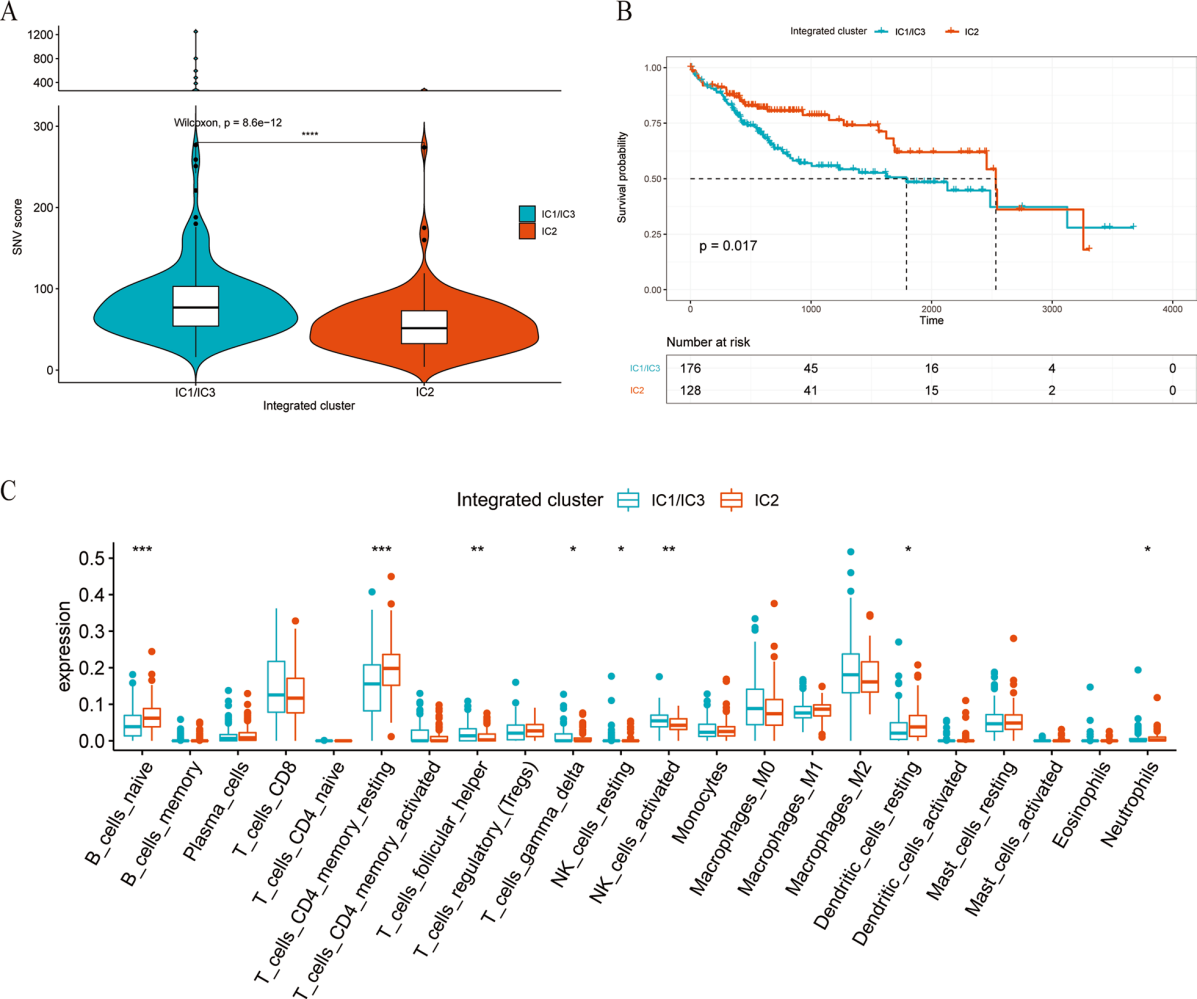
Differences between IC2 and IC1/IC3 (*P < 0.05, **P < 0.01, ***P < 0.001, ****P < 0.0001, ns means no statistical significance, according to two independent samples with a Wilcox test): **A** Dimensions of the SNV score. **B** KM curve analysis in two subgroups after optimization. C, Abundances of twenty-two kinds of immune cells in IC1/IC3 and IC2

To further optimize the integrated clusters, we combined IC1 and IC3 into a new subgroup, called IC1/IC3, with similar SNV scores and clinical characteristics. The SNV score of IC1/IC3 was higher than that of IC2 (P < 0.0001, Fig. 2A), and their prognosis was worse than that of IC2 (P = 0.017, Fig. 2B). There were no significant differences in clinical features between IC1/IC3 and IC2 (Table 1).

**Table 1:** Pathological and clinical features of the integrated clusters after optimization

		IC1/IC3 (n = 176)	IC 2 (n = 128)		P
Pathologic stage	1 + 2	126	101	*χ*^*2*^ = 2.097	0.1476
	3 + 4	50	27		
Pathologic T	1 + 2	127	102	*χ*^*2*^ = 2.260	0.3230
	3 + 4	44	21		
Gender	Female	52	43	*χ*^*2*^ = 0.5653	0.4521
	Male	124	85		
BMI		25.40 ± 5.762	26.95 ± 11.39	*t* = 1.557	0.1206

We evaluated the infiltration of immune cells between IC1/IC3 and IC2 using the algorithm called cibersoft. The resting CD4 T cells and resting dendritic cells of IC2 were higher than those of IC1/IC3, while the follicular helper T cells and activated NK cells were lower than those of IC1/IC3 (Fig. 2C).

### Differentially expressed genes between IC1/IC3 and IC2 mainly play a role in the extracellular matrix by GO analysis and can be mapped to the PI3K-Akt signalling pathway by KEGG

**Fig. 3:**
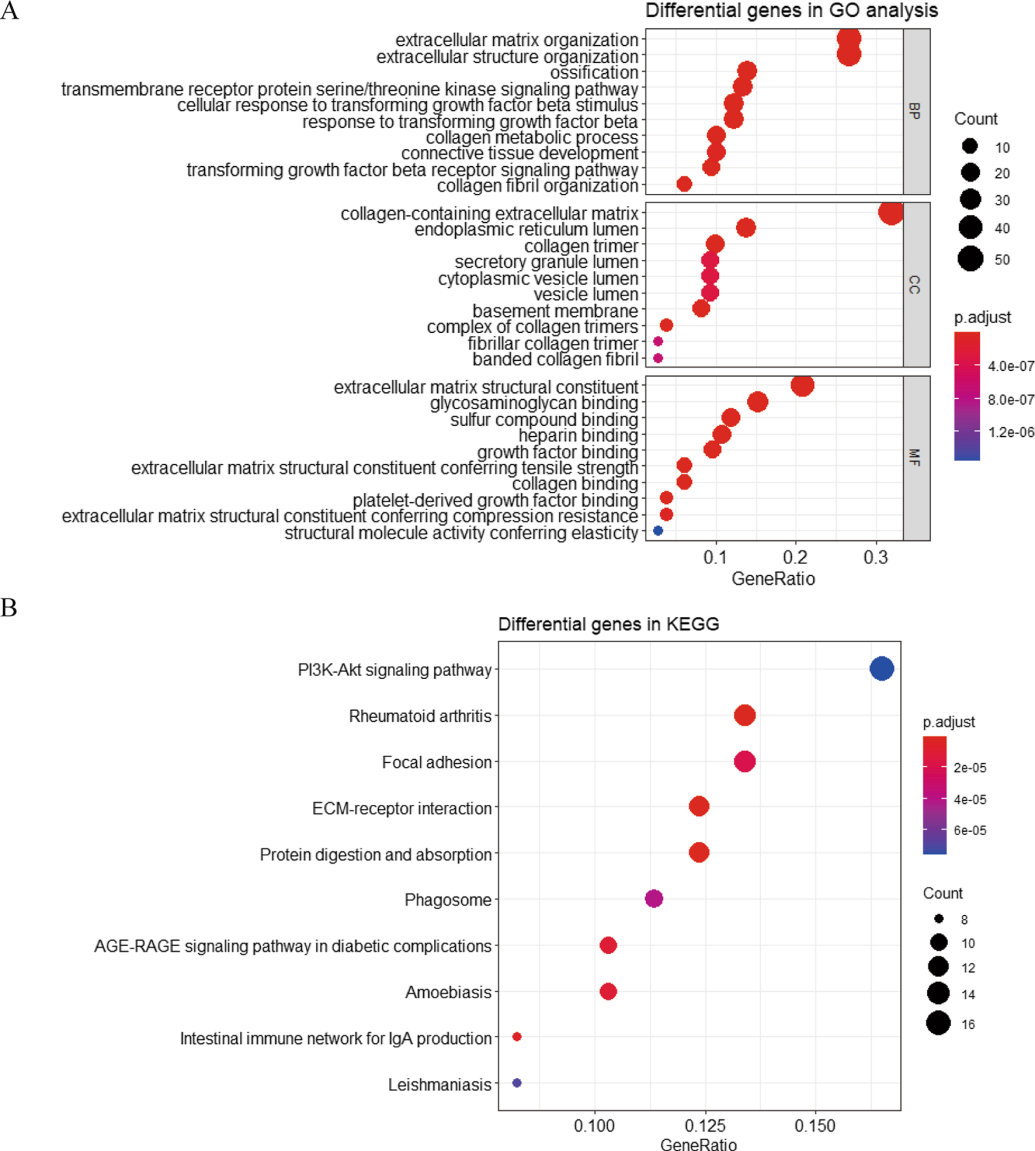
GO and KEGG analyses of differentially expressed genes between IC1/IC3 and IC2: **A** Differential expressed genes between IC1/IC3 and IC2 were performed into GO analysis including BP, CC and MF dimensions.(the larger size of the cycles represents the more count of genes mapped into the pathway or biological process, the color of the cycles from blue to red represents the adjust P value from high to low); **B** KEGG analyses of differentially expressed genes between IC1/IC3 and IC2 (the larger size of the cycles represents the more count of genes mapped into the signalling pathway, the color of the cycles from blue to red represents the adjust P value from high to low)

According to the optimized integrated clusters, we screened 182 differentially expressed genes between IC1/IC3 and IC2. The results of GO analysis showed that these genes were significantly concentrated in the extracellular matrix organization and extracellular structure organization in the biological process (BP) dimension. In the cellular component (CC) dimension, they were mainly concentrated in the collagen-containing extracellular matrix, while in the molecular function (MF) dimension, they were mainly concentrated in the structural flow of the extracellular matrix (Fig. 3A). In KEGG analysis, the PI3K-Akt signalling pathway was identified as the top pathway with the richest differential expressed genes and the lowest adjusted P value (Fig. 3B).

### Survival model construction based on seven key differentially expressed genes

**Fig. 4:**
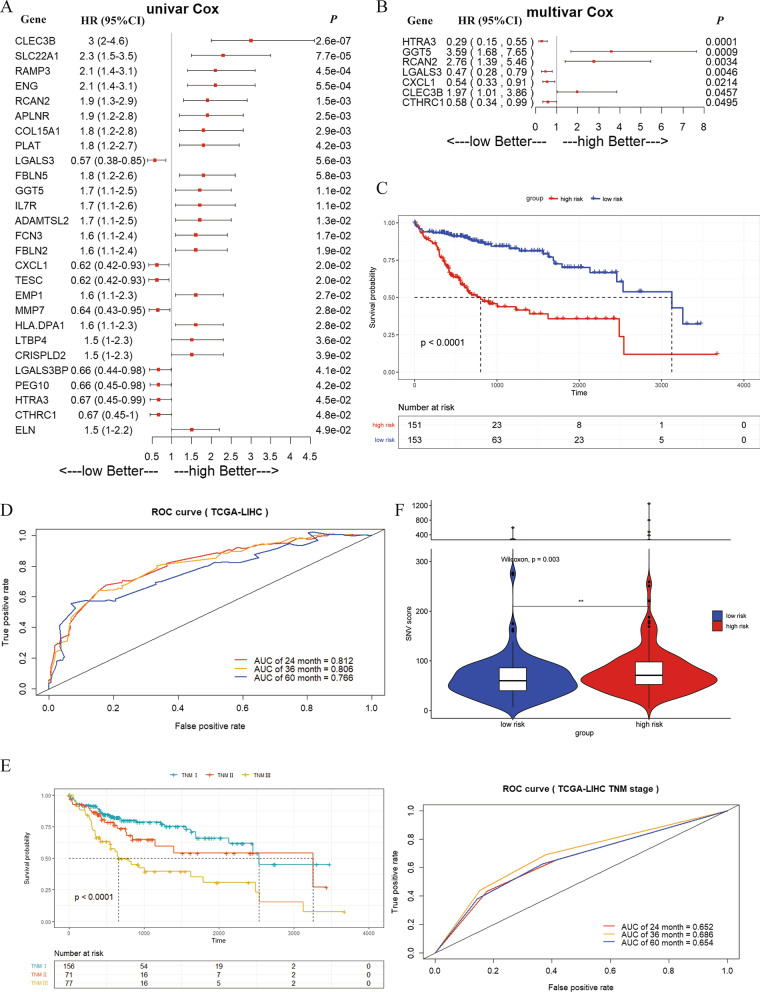
Construction of the survival model in the TCGA-LIHC dataset: **A** 182 differentially expressed genes between IC1/IC3 and IC2 were imported into the univariate analysis workflow of the Cox proportional hazard model and finally twenty-seven genes with P < 0.05 were accessed. **B** Twenty-seven genes with log-rank P < 0.05 in Cox univariate hazard analysis shown in Fig. 4A were selected out for cox multivariate hazard analysis and finally we got seven genes with P < 0.05 in Cox multivariate hazard analysis. **C** The KM curve in high-risk and low-risk groups. **D** ROC curve analysis at 24, 36, and 60 months. **E** The KM curve and ROC curve analysis of TNM stage in TCGA-LIHC database. F, The SNV score in different risk groups (*P < 0.05, **P < 0.01, ***P < 0.001, ****P < 0.0001, ns means no statistical significance, with a Wilcox test)

The differentially expressed genes were imported into the univariate and multivariate analysis workflow of the Cox proportional hazard model. In the section of univariate analysis, we calculated the log-rank P between each gene and overall survival one by one and finally accessed 27 genes with log-rank P < 0.05 as the significant standard (Fig. 4A). Subsequently, 27 genes we accessed from univariate analysis were imported together into the workflow of multivariate analysis (P < 0.05 was set as the threshold). Finally, seven genes were identified as independent factors of overall survival and were used to constructed a survival model (Fig. 4B). The correlation coefficients and P value of these seven key differentially expressed genes were shown in Additional file 1: Table S2. Most of them have the P < 0.05 while only GGT5 meets the criterion of SNV score-related genes.

Based on the survival model, each patient’s risk score was calculated by the following formula: risk score = (coef_HTRA3 × HTRA3_low) + (coef_GGT5 × GGT5_low) + (coef_RCAN2 × RCAN2_low) + (coef_LGALS3 × LGALS3_low) + (coef_CXCL1 × CXCL1_low) + (coef_CLEC3B × CLEC3B_low) + (coef_CTHRC1 × CTHRC1_low). Gene_low represents the gene expression in the low expression group and was assigned a value of 1.0; otherwise, it was assigned a value of 0. The coefficient of each gene in the risk prediction formula of the survival model is shown in Table 2.

**Table 2:** The coefficient of each gene in COX proportional hazard model

Gene	Coefficient
*HTRA3*	− 1.223
*GGT5*	1.278
*RCAN2*	1.016
*LGALS3*	− 0.744
*CXCL1*	− 0.598
*CLEC3B*	0.682
*CTHRC1*	− 0.538

With a risk score cut-off of 1.0, the patients were divided into high-risk and low-risk groups. The KM survival curves were used to evaluate this new survival model and they showed that the survival in the high-risk group was worse than that in the low-risk group (log-rank *P* < 0.0001, Fig. 4C). As shown in Fig. 4D, the AUC values were 0.812, 0.806 and 0.766 in ROC curve analysis at 24, 36 and 60 months, respectively, suggesting that the model has good specificity and sensitivity for prognostication. Compared with the TNM stage, the AUC value of survival model was higher than that of the TMN stage (the AUC values of TNM stage were 0.652, 0.686 and 0.654, Fig. 4E). It seemed that the survival model can provide the more acceptable specificity and sensitivity in prognosis prediction than TNM stage. In addition, the SNV score of the high-risk group was higher than that of the low-risk group (P = 0.003, Fig. 4F).

### Two independent external test datasets verified the SNV score-related genes-based model to be an effective method to predict the outcomes of the HCC patients

**Fig. 5:**
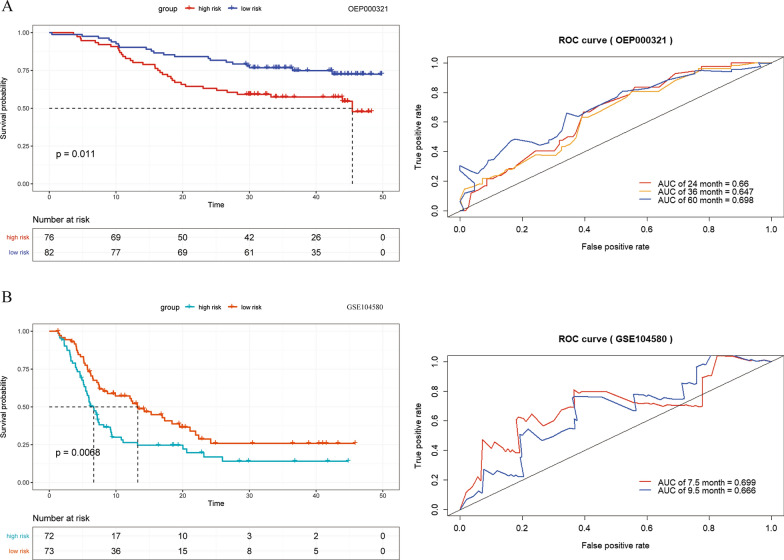
Validation in two external test datasets: **A** The KM and ROC curve analyses in OEP000321. **B** The KM and ROC curve analyses in GSE104580

In the first test dataset (OEP000321), we identified 76 patients in the high-risk group and 82 patients in the low-risk group using the same risk score risk cut-off as the training dataset. The overall survival of the high-risk group was worse than that of the low-risk group, with an acceptable specificity and sensitivity (log-rank P = 0.011, AUC = 0.66, 0.647, and 0.698 at 24, 36, and 60 months, respectively, Fig. 5A).

To increase the reliability of the model, we used another external test dataset (GSE104580) to conduct a second validation. All patients in GSE104580 were diagnosed with unresectable HCC in an advanced stage and were treated with TACE therapy instead. The baseline clinical features in GSE104580 and the results of the Cox univariate analysis are shown in Additional file 1: Table S3.

The median survival time in GSE104580 was 7.23 months. The results of the KM survival curves showed that the overall survival of the 73 patients in the low-risk group was better than that of the 72 patients in the high-risk group (log-rank P = 0.0068, Fig. 5B). The AUC values were 0.699 and 0.666 when 7.5 and 9.5 months were taken as checkpoints for the ROC curve analysis. The risk score of the model, together with age, tumour size, vascular invasion and metastasis, was one of the independent risk factors affecting prognosis (Table 3).

**Table 3:** Cox multivariate analysis in GSE104580

	HR (95%CI)	*P*
Risk	1.59 (1.07–2.38)	0.023
Age	1.67 (1.10–2.55)	0.016
Size	0.58 (0.36–0.91)	0.019
Vascular invasion	1.69 (1.13–2.52)	0.011
Metastasis	2.29 (1.19–4.41)	0.013

### The regulator of calcineurin 2 (RCAN2) was inversely correlated with NK cell infiltration, and knockdown of RCAN2 promoted proliferation in HCC

RCAN2, encoding a member of the regulator of calcineurin protein family, regulates the level of phosphorylation by binding to the catalytic domain of calcineurin A. In KEGG analysis, differentially expressed genes were mapped to the PI3K-Akt signalling pathway, and phosphorylation plays an important role in this process. On this basis, RCAN2 was screened out as a key gene for validation in vitro.

**Fig. 6:**
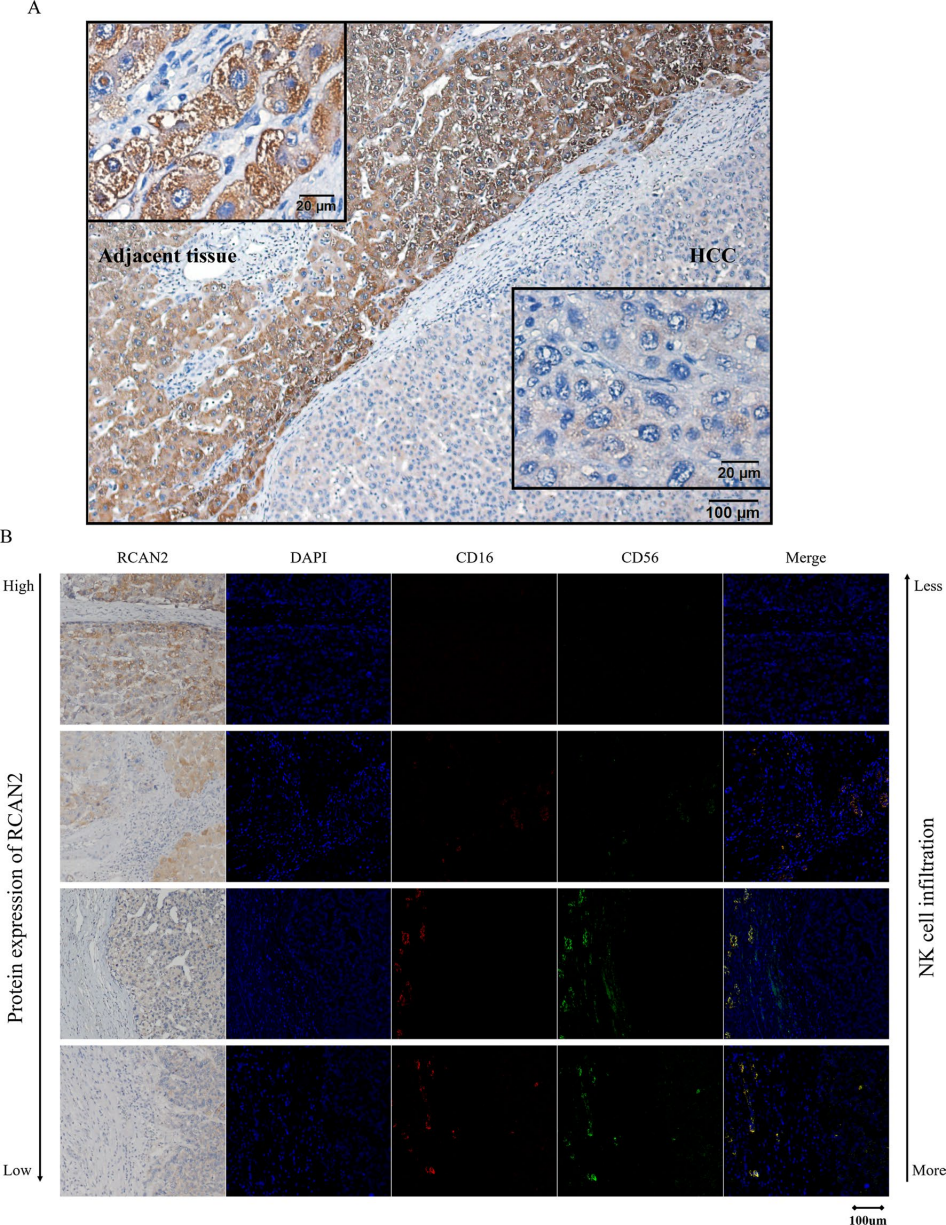
IHC and multicolour IF in clinical tissue of HCC: **A** Characteristics of RCAN2 protein expression. **B** NK cell infiltration in HCC tissues with different RCAN2 protein expression levels

The RCAN2 protein was located in the cytoplasm in HCC and in adjacent tissue. As shown in Fig. 6A, the expression level of RCAN2 in HCC was lower than that in adjacent tissue. CD16 and CD56 were stained to detect NK cells in HCC by multicolour immunofluorescence, and it seemed that RCAN2 expression was inversely correlated with NK cell infiltration (Fig. 6B).

**Fig. 7:**
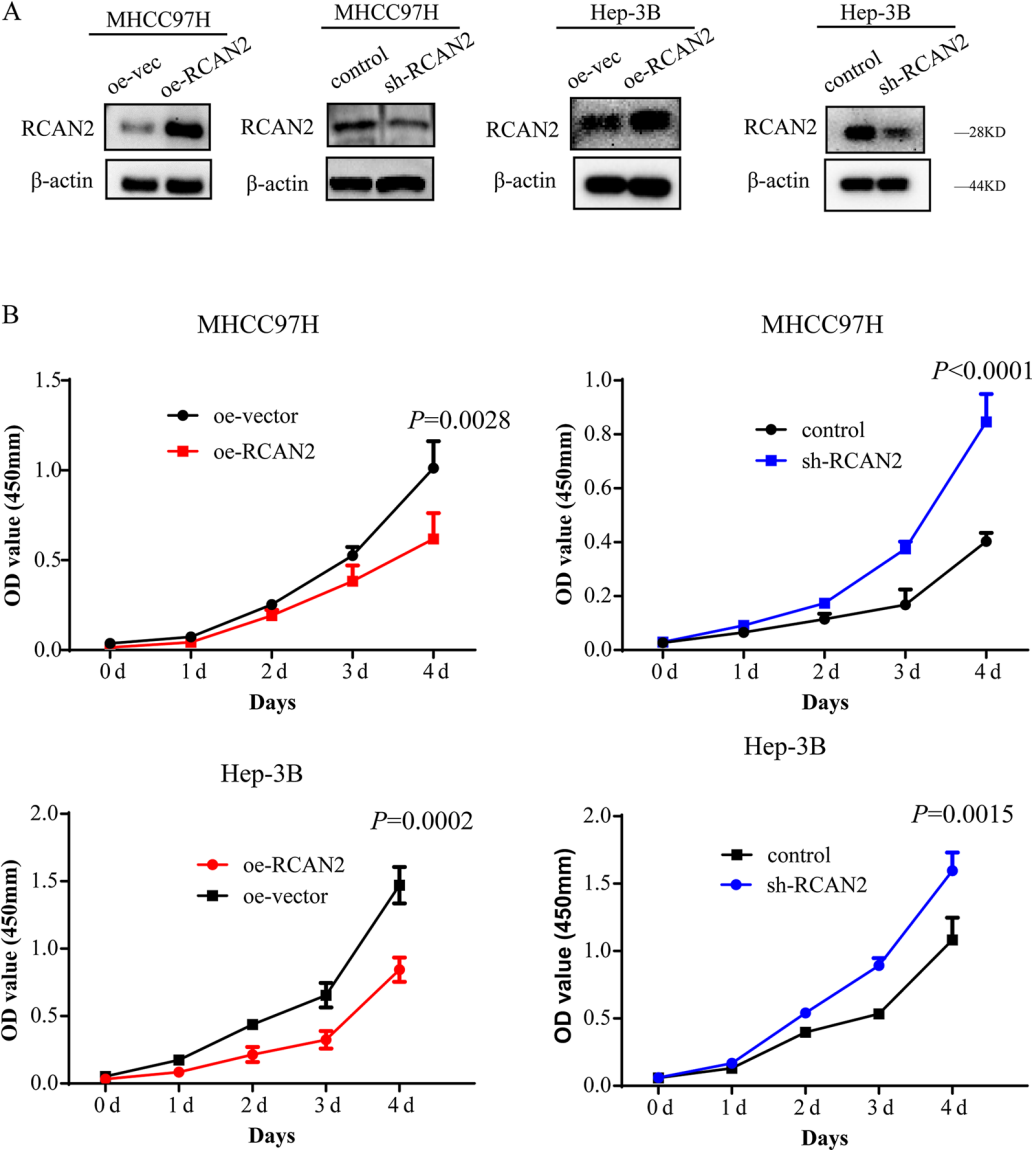
Validation of RCAN2 in vitro: **A** Overexpression and knockdown of RCAN2 in MHCC97H and Hep-3B cells. **B** The proliferation of these cells

We overexpressed and knocked down RCAN2 in MHCC97H and Hep3B cells (Fig. 7A). To examine whether the proliferation of HCC is associated with RCAN2, we measured cell proliferation by examining the optical density at 450 nm with a CCK8 assay. Obviously increased cell proliferation was revealed in MHCC97H and Hep3B cells after knocking down RCAN2, while the proliferation rate was decreased when RCAN2 was overexpressed (Fig. 7B).

## Discussion

With the accumulation of gene mutations in HCC, its malignant degree and invasion risk are also increasing [7]. Within the same period, mutations also lead to the emergence of tumour-specific neoantigens, which may improve the efficacy of immunotherapy by enhancing the immune recognition of tumours [22]. Therefore, gene mutations have a complex and multidimensional influence on the prognosis of HCC. Considered one of the important gene mutations, SNVs play an important role in the development and prognosis of HCC.

To further explore the mechanism through which SNV influences HCC, the differentially expressed genes between IC1/IC3 and IC2 were analysed by GO and KEGG analyses. These results showed that the differentially expressed genes were mainly located in the extracellular matrix and might play a biological function through signal transmission between tumour cells and the tumour microenvironment. In addition, the KEGG results suggested that the accumulation of SNVs affected cellular functions through the PI3K-Akt signalling pathway, leading to an ultimate difference in the survival of patients with HCC.

Although the importance of SNVs has been gradually revealed, their value in clinical transformation has rarely been assessed. We screened seven key genes, including HTRA3, GGT5, RCAN2, LGALS3, CXCL1, CLEC3B and CTHRC1, from among the differentially expressed genes by Cox hazard analysis. We further established a survival prediction model of HCC with the seven screened-out key genes. Our results indicated that GGT5, RCAN2 and CLEC3B were positively related to patient prognosis, while the others were negative indicators. This is consistent with previous studies showing that all of these genes were closely related to tumour proliferation and the immune microenvironment in various cancer types [23–29]. The specificity and sensitivity of this model were examined with two independent datasets. ROC curve analysis showed an acceptable AUC value in the two external test datasets.

Interestingly, there was a higher SNV score in IC1/IC3 than in IC2. Meanwhile, NK cells were significantly higher in IC1/IC3 than in IC2. Our study of RCAN2, the key gene selected from among the differentially expressed genes, also obtained similar results in vitro: knockdown of RCAN2 could increase the growth rate of HCC cells, and the protein expression of RCAN2 was negatively correlated with the infiltration of NK cells. Although the use of tumour mutation load to predict the overall survival of patients with cancer remains controversial, patients with a high tumour mutation load can benefit from immunotherapy and have a better objective response rate in melanoma, lung, and bladder cancers [30, 31]. In TCGA-LIHC, the predictive model results were closely related to the tumour mutation load. It seemed that for patients in the high-risk group identified by the model, combined immunotherapy may be one of the valuable research directions. However, further confirmation by corresponding clinical studies is still needed. For the above reasons, a clinical study on the effect and safety of HAIC combined with PD1 in patients with advanced HCC was designed and promoted by our team (NCT04135690).

The shortcomings of this study lie in the lack of whole genome or exon sequencing data in the test datasets, which makes it impossible to further evaluate whether high-risk patients identified by the model are associated with SNVs or gene mutations. In the future, the model can be modified by adding other external validation datasets. Although there are some limitations at present, these key genes related to SNVs and survival can be used as biomarkers for HCC reclassification. More importantly, the survival model provides a new perspective and strategy for personalized therapy of HCC.

## Conclusions

We identified HTRA3, GGT5, RCAN2, LGALS3, CXCL1, CLEC3B and CTHRC1 as key genes that were expressed in parallel with SNVs and predicted overall survival. It was preliminarily proven in vitro that the expression of RCAN2 could influence proliferation in HCC and had a close relationship with NK cell infiltration. The survival model constructed with seven key genes had acceptable accuracy, sensitivity and specificity, as verified in two independent external test databases, and its results were closely related to the tumour mutation load. This study provides a new perspective and basis for HCC immunotherapy.

## Supplementary Information

**Additional file 1:** **Table S1.** Pathological and clinical features of the original integrated clusters. **Table S2.** The correlation coefficients and P value between these seven key differentially expressed genes and SNV score. **Table S3.** The baseline of clinical features in GSE104580 and the cox univariate analysis.

## Data Availability

The training datasets were generated from The Cancer Genome Atlas-Liver Hepatocellular Carcinoma (TCGA-LIHC) database (https://portal.gdc.cancer.gov). The external test database is accessible in NODE (https://www.biosino.org/node, ID# OEP000321) and Gene Expression Omnibus (https://www.ncbi.nlm.nih.gov/geo, ID: GSE104580). MHCC97H cell was obtained at Liver Cancer Institute, Fudan University. Hep3B cell was purchased from the National Collection of Authenticated Cell Cultures (https://www.cellbank.org.cn/).

## Abbreviations

HCC: Hepatocellular carcinoma
DNA: Deoxyribo nucleic acid
SNV: Single nucleotide variant
TCGA-LIHC: The Cancer Genome Atlas- Liver hepatocellular carcinoma
IC: Integrated cluster
NK cell: NK cell
GO: Gene Ontology
KEGG: Kyoto Encyclopedia of Genes and Genomes
KM: Kaplan–Meier
ROC: Receiver operating characteristic
AUC: Area under curve
TACE: Transcatheter arterial chemoembolization
IHC: Immunohistochemistry
IF: Immunofluorescence
CCK8: Cell counting kit-8
BMI: Body mass index
BP: Biological process
CC: Cellular component
MF: Molecular function
HTRA3: HtrA: serine peptidase 3
GGT5: Gamma-glutamyltransferase 5
RCAN2: Regulator of calcineurin 2
LGALS3: Galectin 3
CXCL1: C–X–C motif chemokine ligand 1
CLEC3B: C-type lectin domain family 3 member B
CTHRC1: Collagen triple helix repeat containing 1
