# Supplementary material for: Construction of a single nucleotide variant score-related gene-based prognostic model in hepatocellular carcinoma: analysis of multi-independent databases and validation in vitro
Source: Cancer Cell Int. 2021 Nov 18;21:610. doi: 10.1186/s12935-021-02321-z (PMC8600893; doi:10.1186/s12935-021-02321-z)
Supplement: Supplementary file 1 — Additional file 1: Table S1. Pathological and clinical features of the original integrated clusters. Table S2. The correlation coefficients and P value between these seven key differentially expressed genes and SNV score. Table S3. The baseline of clinical features in GSE104580 and the cox univariate analysis. [file 12935_2021_2321_MOESM1_ESM.docx]

Supplemental table 1. Pathological and clinical features of the original integrated clusters.

|  |  | IC1 (n=161) | IC2 (n=128) | IC3 (n=15) |  | *P* |
| --- | --- | --- | --- | --- | --- | --- |
| Pathologic stage | 1+2 | 118 | 101 | 8 | *Kruskal-Wallis=*4.97 | 0.083 |
|  | 3+4 | 43 | 27 | 7 |  |  |
| Pathologic T | 1+2 | 118 | 102 | 9 | *Kruskal-Wallis=*3.55 | 0.169 |
|  | 3+4 | 43 | 26 | 6 |  |  |
| gender | female | 46 | 43 | 6 | *Kruskal-Wallis=*1.34 | 0.498 |
|  | male | 115 | 85 | 9 |  |  |
| BMI |  | 25.47±5.921 | 26.95±11.39 | 24.62±3.679 | *F=* 1.28 | 0.281 |

Supplemental table 2. The correlation coefficients and P value between these seven key differentially expressed genes and SNV score

| Gene symbol | Correlation coefficients | P value |
| --- | --- | --- |
| HTRA3 | -0.142 | 0.007 |
| GGT5 | -0.204 | 0.0001 |
| RCAN2 | -0.115 | 0.031 |
| LGALS3 | -0.034 | 0.520 |
| CXCL1 | -0.056 | 0.299 |
| CLEC3B | -0.177 | 0.0008 |
| CTHRC1 | -0.123 | 0.022 |

Supplemental table 3. The baseline of clinical features in GSE104580 and the cox univariate analysis.

|  | Low (N=73) | High (n=72) | *P* | univariate analysis | |
| --- | --- | --- | --- | --- | --- |
|  |  |  |  | OS | *P* |
| Age |  |  | 0.20 |  | 0.02 |
| ≦50y | 39 | 46 |  | 7.1 |  |
| ＞50y | 34 | 26 |  | 12.3 |  |
| Gender |  |  | 0.73 |  | 0.53 |
| Female | 5 | 3 |  | 12.3 |  |
| Male | 68 | 69 |  | 7.5 |  |
| Size |  |  | 0.005 |  | 0.003 |
| ≦10cm | 30 | 14 |  | 16.9 |  |
| ＞10cm | 43 | 58 |  | 6.7 |  |
| AFP |  |  | 0.08 |  | 0.01 |
| ≤400ng/ml | 37 | 26 |  | 13.2 |  |
| ＞400ng/ml | 36 | 46 |  | 6.7 |  |
| Tumour number |  |  | 0.54 |  | 0.05 |
| ≤3 | 55 | 51 |  | 9.9 |  |
| ＞3 | 18 | 21 |  | 6.5 |  |
| Vascular invasion |  |  | 0.79 |  | 0.008 |
| No | 41 | 42 |  | 12.5 |  |
| Yes | 32 | 30 |  | 5.8 |  |
| Metastasis |  |  | 0.74 |  | 0.009 |
| No | 68 | 66 |  | 9.2 |  |
| Yes | 5 | 6 |  | 6.0 |  |
